# Development of Efficient In-Situ Cleaning Methods for Stained Textile Relics

**DOI:** 10.3390/gels11100830

**Published:** 2025-10-16

**Authors:** Yuhui Wei, Jinxia Guo, Zhaowei Su, Kui Yu, Xue Ling, Zhenlin Zhang, Kaixuan Liu, Wei Pan

**Affiliations:** 1 College of Textile and Clothing, Anhui Polytechnic University, Wuhu 241000, China18805569377@139.com (K.Y.);; 2 High Fashion Womenswear Institute, Hangzhou Polytechnic University, Hangzhou 310018, China; 3 College of Physical Education, Anhui Polytechnic University, Wuhu 241000, China; 13866350328@163.com; 4 School of Fashion and Art Design, Xi’an Polytechnic University, Xi’an 710048, China; liukaixuan819@163.com; 5 Anhui Suli Technology Co., Ltd., Wuhu 241000, China

**Keywords:** stained textile relics, in-situ cleaning, cleaning efficiency, gel material, ultrasonic cleaning

## Abstract

To address limitations such as cleaning difficulties or secondary contamination/damage of cultural relics caused by the uncontrollable diffusion of water/cleaning agent/dirty liquids during the cleaning process in traditional cleaning methods, this study, using cotton textiles as an example, systematically investigated the cleaning efficacy of four in situ methods (blank gel, cleaning gel, ultrasonic emulsification, and gel + ultrasonic emulsification synergistic cleaning) on eight types of stains, including sand, clay, rust, blood, ink, oil, and mixed solid/liquid stains. Building upon this, this study proposed an efficient, targeted, in situ, and controllable cleaning strategy tailored for fragile, stained textile relics. Results demonstrated that, regardless of the stain type, the synergistic cleaning method of G+U (gel poultice + ultrasonic emulsification) consistently outperformed the cleaning methods of blank gel poultice, cleaning gel poultice, and ultrasonic emulsification. Furthermore, the gel loaded with cleaning agents was always more effective than the blank gel (unloaded cleaning agents). The poultice methods of blank gel and cleaning gel were better suited for solid stains, while the ultrasonic emulsification cleaning method was more effective for liquid stains. Meanwhile, it was also found that the optimal cleaning method proposed in this study (the G+U synergistic cleaning method) was a cleaning method that restricted the cleaning agent within the gel network/emulsion system, and utilized the porous network physical structure of gel, the chemical action of emulsion’s wetting/dissolving dirt, and the cavitation synergistic effect of ultrasound to achieve the targeted removal of contaminants from relics’ surfaces. Crucially, the cleaning process of G+U also had the characteristics of controlling the cleaning area at the designated position and effectively regulating the diffusion rate of the cleaning solution within the treatment zone, as well as the reaction intensity. Therefore, the proposed optimal (the synergistic cleaning method of G+U) cleaning method conforms to the significant implementation of the “minimal intervention and maximal preservation” principle in modern cultural heritage conservation. Consequently, the synergistic cleaning method of G+U holds promise for practical application in artifact cleaning work.

## 1. Introduction

**Figure 1:**
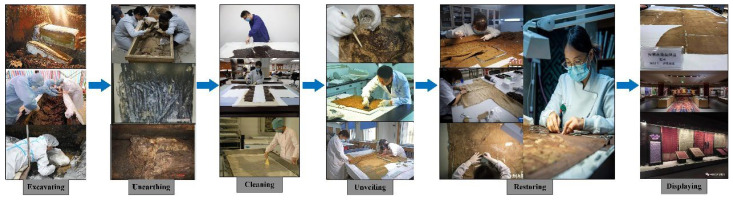
The entire process of textile relic conservation.

Textile relics, with distinctive weaving styles and cultural attributes, form a significant part of China’s five millennia of civilization [1,2,3]. Simultaneously, textile relics are also crucial physical evidence revealing the evolution of ancient textile manufacturing techniques, and are invaluable resources for understanding the cultural, economic, and social environments, as well as human activities of their time [4,5,6]. Consequently, textile relics play a vital role in preserving and transmitting China’s outstanding ancient history and culture. Unfortunately, textile relics are also a category of cultural relics that have been subjected to prolonged erosion by burial environments, including soil, decaying organic matter, burial liquids, and molds [7,8,9]. Upon excavation, they are often found with substantial deposits of soil, decomposed biological matter, mold, corpse fluids, and other stains accumulated on their surfaces or within their structures [10,11,12]. The accumulation and enrichment of these stains on the surface obscure the artifacts’ exquisite patterns and unique colors, diminishing their artistic and aesthetic value. Moreover, if not cleaned promptly, they cause the embedded contaminants (such as soil, burial liquids, and mold) to further blur the artifacts and accelerate the internal deterioration (rotting, embrittlement, carbonization, and decay), thus leading to irreversible damage that is detrimental to artifact preservation [13]. Therefore, cleaning is the primary task in the storage, conservation, and restoration of cultural relics such as textiles (see Figure 1), and a crucial step in determining the feasibility and success of subsequent conservation and restoration efforts [14,15,16].

However, current research on textile relics primarily focuses on aspects such as artifact texture, pattern design, weaving techniques, identification and restoration, reinforcement material development, storage environment analysis, and aging simulation testing [2,10,17,18]. Relatively less research exists on the cleaning of adhered stains on textile relics [14,19]. Simultaneously, the types of stains on textile relics originate from long-term burial in subterranean environments, mainly involving microbial corrosion byproducts, groundwater, soil and its minerals, coffin fluids, mold, and other contaminants [20,21]. In contrast, stains on ordinary clothing/textiles primarily stem from daily life (food residues, human metabolic waste, ink marks, wine, air pollutants, etc.) or workplace contaminants (such as blood or sputum stains in hospitals). Clearly, there is a significant difference in the sources of stains between the two [22,23]. Furthermore, the accumulation time of stains on textile relics is generally far longer than that on everyday textiles/garments [24]. Moreover, studies on the cleaning of textile stains demonstrate that the adsorption and desorption mechanisms of different stains with the substrate vary significantly and, consequently, optimal cleaning methods also differ [25,26,27]. Therefore, the cleaning techniques for stains developed for ordinary textiles/garments are not suitable for textile relics. Additionally, textile relics belong to extremely precious historical artifacts [28,29,30]. Consequently, the cleaning of textile relics must adhere to the conservation principles of “preserving the aged appearance” (“cleaning the old as the old”) and the requirements of “minimal intervention” and “not altering the artifact’s appearance” [3,31]. Moreover, traditional cleaning methods, such as chemical cleaning, laser cleaning, steam cleaning, micro/nanobubble cleaning, and mechanical cleaning, are effective but pose problems, including uncontrollable diffusion and the flow of cleaning solutions, residual cleaning agents, expansion of contaminated areas, and potential degradation or even destruction of the artifact [24,32]. Hence, traditional methods (laser, steam, micro-particle blasting, soaking, mechanical, and chemical cleaning) are generally unsuitable for cleaning textile relics. Conversely, gel materials, as cross-linked polymer networks with unique capillary structures and mechanical properties, can swell in water or organic solvents to form soft matter, endowing them with an excellent liquid retention capacity and adhesion [24,32,33]. Thus, the cleaning method of using gel as a poultice material has become more appropriate for removing stains from textile relics [12,34,35]. Meanwhile, the poultice cleaning method of gel systems also can effectively minimize fluid flow, volatilization, penetration, and the diffusion of cleaning solutions on cultural relics’ surfaces, and enable the targeted, controllable, site-specific, in-situ removal of contaminants [34,35]. In addition, the cleaning method of using gel-based systems also maximizes the reduction in cleaning-induced damage so to protect relics and extend their lifespan [34,35]. However, gel cleaning has mainly been applied in the field of cultural relic conservation, particularly for artifacts such as murals, paper, and wood. There have been no reported studies on the use of gel materials in textile artifact cleaning, let alone systematic comparative analyses of gel cleaning effectiveness for different types of stains. Moreover, textile artifacts are composed of textile fibers, whose internal structure, surface morphology, and types of deposited stains differ significantly from those of murals, paper, wood, and other similar artifacts. Additionally, stain cleaning theory indicates that cleaning effectiveness varies markedly depending on the substrate material and the type of stain. Thus, it is very necessary to carry out research on textile relic cleaning under gel systems.

Therefore, this study proposes using gel as the substrate to conduct simulated cleaning experiments on eight types of common stains on textile relics, including sand, clay, rust, mixed solids (sand–clay–rust), artificial blood, oil, ink, and mixed liquids (artificial blood–ink–oil stains), and systematically investigates the effects of four in situ cleaning methods (pure gel, cleaning gel, ultrasonic microemulsion, and gel combined with ultrasonic microemulsion) on the removal efficiency of eight typical stains. This study also clarifies the cleaning characteristics, reveals the underlying cleaning mechanisms, and thus ultimately develops an optimal in-situ cleaning method suitable for stained textile relics. This study not only expands the fine cleaning technology within textile relic conservation but also enhances the overall framework for the cleaning, protection, and restoration of textile relics. Furthermore, it provides a basis for optimizing and standardizing cleaning procedures for actual textile relics.

## 2. Results and Discussion

In order to develop efficient in-situ cleaning methods for stained textile relics, the appearance morphology, washing efficiency, mechanical strength, and EDS of stained textiles (the simulated samples of stained textile relics) before and after cleaning with different in-situ cleaning methods were analyzed and compared.

### 2.1. Appearance Morphology Analysis of Stained Textile Relics Before and After Cleaning

**Figure 2:**
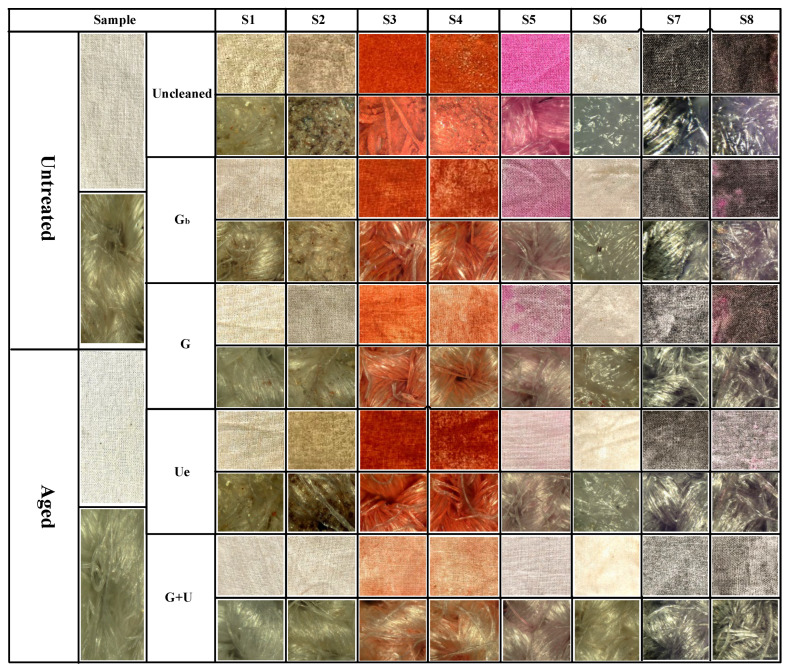
Appearance morphology of stained textiles before and after cleaning with different methods.

Figure 2 clearly shows the morphological alterations in the appearance of stained textiles before and after cleaning with different in-situ cleaning methods. As illustrated in Figure 2, regardless of the stain type, stains adhering to textiles were reduced to some extent after cleaning treatments, but the removal effectiveness varied depending on the stain type and cleaning method compared to the uncleaned state. Specifically, for textiles adhered with solid stains, regardless of the stain type, after cleaning with blank gel (unloaded cleaning agent) or cleaning gel (loaded with cleaning agent) poultice treatments, the granular stains on the textile surfaces were significantly reduced. However, stains within the fiber gaps/internal structure were still clearly visible. Furthermore, stain removal exhibited an uneven, speckled flaking phenomenon. This is because the poultice cleaning methods using blank gel and cleaning gel are essentially based on solid-phase extraction technology. They utilize the strong adhesion of the gel to selectively bind target molecules (stains) onto the three-dimensional network structure of the solid-phase carrier (gel), thereby separating stains from the substrate (textiles). Consequently, these gel poultice methods can only remove granular stains adhering to the yarn or textile surface but not those penetrating the fiber interior. Additionally, it was observed that the reduction in surface stain granularity for stained textiles/yarns treated with the blank gel poultice was less than that for textiles treated with the cleaning gel poultice. This indicated that the blank gel had inferior stain removal effectiveness compared to the cleaning gel. This difference occurred because the blank gel poultice relied solely on adsorption, whereas the cleaning gel poultice combined adsorption with the chemical action of detergent components. Therefore, the cleaning effect of blank gel poultice was inferior to that of cleaning gel. This also demonstrated that the poultice method of blank gel primarily represented a change in the cleaning vehicle, thus improving the handling of the solution rather than altering the primary active cleaning ingredients. Thus, to effectively and precisely remove stains using this approach in the future, it must be combined with compatible cleaning agents to achieve the desired cleaning results. Conversely, stained textiles cleaned with ultrasonic emulsification showed a significant reduction in granular surface stains, but the chromaticity of stained textiles appeared similar to, or even darker than, that of the stained (uncleaned) textiles. This occurred because solid stains adhered to textile surfaces dissolved upon encountering the dense cleaning emulsion and were subsequently deposited deeper into the fiber structure, resulting in insignificant or even increased apparent staining. In contrast, stained textiles treated with the combined cleaning gel poultice + ultrasonic emulsification method exhibited a significant reduction in deposited stains on the textile surfaces, within the fiber interiors, and in the crevices. Textiles adhered with sand/clay stains almost recovered to their original color (oatmeal). Textiles stained with rust/mixed solid stains, although not fully restored to their original color, showed a significant decrease in the degree of contamination.

Furthermore, comparing different stains revealed that, under the same cleaning method, the reduction in surface particles was most pronounced for sand-adhered textiles/fibers, followed by clay and mixed solid stains, with rust showing the least reduction. This indicated that rust was the most difficult to remove, followed by mixed solids and clay, while sand was the easiest to clean. Consequently, cleaning parameters (e.g., cleaning time for difficult stains under the same method should be appropriately extended compared to easy stains) should be rationally designed based on the specific stain type in the actual cleaning and protection work of textile relics. This difference in cleanability stemmed from the nature of the stains: clay and sand primarily consist of mineral and organic physical particles. They adhere to surfaces via gravity, electrostatic adsorption, mechanical interlocking (lodged in fiber or surface gaps), or moisture-induced stickiness without undergoing chemical reactions with the substrate (i.e., textiles); that is, they are physically adhered stains. Rust, however, is a chemical transformation product formed through the reaction of iron (Fe) with oxygen and water (i.e., oxidation), primarily resulting in iron (III) oxide. Rust stains not only adhered to the surface but also penetrated into the porous substrate’s pores and even formed chemical interactions (e.g., complexation and adsorption) with the substrate surface, making them chemically transformed stains that were significantly harder to remove.

For textiles contaminated with liquid stains, regardless of the stain type, the cleaning effectiveness of the poultice cleaning method of blank gel and cleaning gel was relatively poor, especially the blank gel poultice cleaning method. This is because liquid stains penetrated into the fiber interior, and the cleaning mechanism of gel adhesion relies solely on solid-phase extraction to peel stains from the substrate, resulting in unsatisfactory cleaning. Conversely, ultrasonic emulsification cleaning (U_e_) and the combined method of cleaning gel poultice + ultrasonic emulsification (G+U) were more effective when removing stains, particularly the combined cleaning gel poultice + ultrasonic emulsification approach. This is because the ultrasonic emulsification cleaning method can utilize the powerful physical forces (micro-jets and shock waves) generated by ultrasonic cavitation effects to dislodge and break apart the soil (especially solid particles). Simultaneously, this accelerated the penetration, wetting, emulsification, and dispersion processes of emulsifiers/surfactants in the cleaning solution onto oily stains, thereby encapsulating the stain into stable micro-droplets suspended in the cleaning fluid that were then carried away. Clearly, the G_b_ method is a cleaning method that tightly integrates physical (ultrasonic) and chemical (emulsification) actions, enabling the efficient removal of liquid-type stains. However, the combined cleaning gel poultice + ultrasonic emulsification (G+U) method leveraged the synergistic effect of chemical pre-treatment (softening the soil via the cleaning gel) and physical cavitation (dislodging and enhancing emulsification/dispersion). Therefore, the cleaning method of G+U achieved a highly efficient, deep, and thorough removal of stubborn stains while preventing their re-deposition. Thus, the cleaning method of G+U had superior cleaning effectiveness compared to ultrasonic emulsification cleaning alone. Furthermore, a comparison of different stains under identical cleaning conditions revealed that ink stains were the most difficult to clean, followed by mixed stains, then oil stains, with blood stains being the easiest to remove. This is because ink stains have an extremely complex composition (simultaneously containing pigments, solvents, resins, and additives), exhibit strong penetration (into the fiber interior), and, upon drying/curing, form a tough film that encapsulates the pigments and adheres firmly to the fibers. This made the stain highly insoluble yet prone to smearing, rendering ink-stained textiles the most challenging to clean. Artificial blood stains primarily consisted of water, water-soluble red dyes (food coloring, acid dyes, etc.), thickeners (such as corn syrup, carboxymethyl cellulose, and carbomer), and preservatives. Cleaning agents easily dispersed the pigments, removed sugars and thickeners, and broke their chromophores (porphyrin rings), making them relatively easy to clean. Oil stains mainly comprised greases that adhered physically to the textile’s surface. They were insoluble in water but readily soluble in organic solvents or emulsifiable by surfactants. However, the oil stains studied here were oil stains that were retained for one month. The oil penetrated deeper into the fibers and, during exposure, became oxidized, polymerized, or adsorbed dust, turning into viscous, insoluble, stubborn stains. Consequently, the cleaning difficulty of oil stains was slightly higher.

Furthermore, comparing different cleaning methods revealed that textiles stained with solid stains consistently demonstrated significantly better cleaning effectiveness than those stained with liquid stains after poultice cleaning with either blank gel adhesion or cleaning gel. Conversely, for the ultrasonic emulsification cleaning method, textiles stained with solid stains showed inferior cleaning effectiveness compared to those stained with liquid stains. This indicates that the gel application method is more suitable for solid stains, while the cleaning method of ultrasonic emulsification is more effective for liquid stains. Additionally, it was found that, regardless of the stain type, contaminated textiles cleaned with the combined cleaning method of G+U consistently exhibited the greatest reduction in stains, representing the optimal cleaning effectiveness among the four in-situ methods proposed in this study. This superiority arises because the combined method leverages both the chemical effects of cleaning gel (dissolving, decomposing, softening, and wetting the soil, thereby weakening its structure and adhesion to the substrate) and physical effects of ultrasonic emulsification (cavitation impact, peeling, and forceful removal of the weakened soil), thus resulting in the best overall cleaning performance.

### 2.2. Cleaning Efficiency Analysis of Stained Textile Relics Before and After Cleaning

Table 1 clearly presents the whiteness difference (WD) and cleaning rate (CR, %) of stained textiles before and after cleaning using different methods. As shown in Table 1, regardless of the cleaning method or stain type, the whiteness values of the stained textiles changed to some degree after cleaning treatment, but the magnitude of change varied depending on the stain type and cleaning method compared to the pre-cleaning state. Specifically, for stained textiles, regardless of the stain type, the whiteness values increased after poultice cleaning with blank gel/cleaning gel. The increase was greater for textiles stained with solid stains than for those stained with liquid stains, particularly for textiles stained with sand/clay. This indicates that the gel cleaning method is more suitable for solid stains than liquid stains. Reflecting this in the cleaning rate, textiles stained with liquid stains and treated with the gel patch method generally exhibited lower CR values than those stained with solid stains. This is because the cleaning mechanism of the gel poultice cleaning method primarily relies on physical adsorption to separate surface contaminants from the substrate (textiles). It is therefore more effective for removing solid stains (deposited on the surface of the textile/fiber) compared to liquid stains (which penetrate into the fiber interior). Furthermore, it was observed that the increase in whiteness for stained textiles treated with blank gel was smaller than for those treated with cleaning gel. This is because the poultice cleaning method of blank gel relies solely on the gel’s adsorption effect, whereas the poultice cleaning method of cleaning gel leverages both the gel’s adsorption and the chemical action of the detergent’s active components. Consequently, the whiteness change amplitude for the blank gel treatment was less than that for the cleaning gel; that is, the cleaning rate of the poultice cleaning method of blank gel was lower than that of cleaning gel, especially for rust/mixed solid stains. This also demonstrated that the gel primarily served as a carrier, functioning to confine the solution flow rather than acting as the main active cleaning component. For efficient and targeted stain removal by using this method, it must be combined with compatible cleaning agents to achieve the desired cleaning outcome. For stained textiles treated with the ultrasonic emulsification cleaning method, those stained with solid stains exhibited a decrease rather than an increase in whiteness. Conversely, textiles stained with liquid stains showed an increase in whiteness after ultrasonic emulsification cleaning. This indicated that the ultrasonic emulsification method was more suitable for textiles stained with liquid stains or those without surface-deposited solid particles. The decrease in whiteness for textiles absorbing sand/clay occurred because the solid stains on the textile surfaces were dissolved during ultrasonic emulsification and subsequently penetrated further into the fiber interior. However, stained textiles treated with either ultrasonic emulsification alone or the combined gel + ultrasonic emulsification method showed a significant increase in whiteness regardless of the stain type. Furthermore, the increase in whiteness was greater than that observed with the other three cleaning methods. This demonstrated that both the ultrasonic emulsification method and the combined gel + ultrasonic emulsification method were suitable for all stain types and achieved a relatively superior cleaning effect. This enhanced performance was attributed to the mechanism of the combined cleaning gel poultice + ultrasonic emulsification method. First, the cleaning gel patch dissolved, decomposed, softened, and wetted the deposited soil on the textile surfaces, thus weakening its structure and reducing its adhesion to the substrate. It also removed loosely attached or excess soil. Subsequently, the ultrasonic emulsification employed cavitation impact, mechanical peeling, and forceful removal to eliminate the remaining strongly adhered or internalized contaminants within the fibers. Clearly, this combined approach integrated chemical action (emulsification by the detergent’s active components in the gel) with physical action (ultrasonic vibration). It effectively removed both surface-deposited stains and contaminants that had penetrated into the fiber interior and crevices, resulting in optimal cleaning efficiency.

Furthermore, in comparing different stains, it was found that, regardless of the cleaning method, the whiteness of the textiles soiled with adsorbed oil was always higher than that of unsoiled textiles but lower than that of soiled textiles before cleaning. This occurred because the adsorption of oil formed a milky protective film on the textile surfaces, which possessed a certain degree of light reflectivity, thus increasing its whiteness compared to unsoiled textiles. This conclusion also indicated that cleaning treatments could partially restore the original whiteness of oil-adhered textiles. Conversely, for all other stain types, the whiteness after cleaning was always greater than that of stained, uncleaned textiles, but less than that of unstained (aged) textiles. This finding highlighted the distinct characteristics of different stains, demonstrating that cleaning effectiveness required multiple indicators beyond whiteness values. Therefore, subsequent practical assessments of artifact cleaning efficacy should integrate both instrumental (objective) and subjective (photographic/rating) evaluations. Additionally, it was found that, under identical cleaning conditions, the cleaning efficiency for textiles soiled with solid stains consistently followed the order sand > clay > mixed solids > rust. For textiles soiled with liquid stains, the cleaning efficiency consistently followed the order blood > oil > ink > mixed liquids. This indicated that, among solid stains, rust was the most difficult to remove, followed by mixed stains, then clay, with sand being the easiest to clean. Consequently, for cleaning actual relics, cleaning parameters should be rationally designed based on the specific stain type (e.g., extending the cleaning time for difficult-to-remove stains under the same cleaning method compared to easily removable ones). This difference arose because clay and sand primarily consisted of physical particle types (minerals, organics, etc.) adhering to the surface via gravity, electrostatic attraction, mechanical interlocking (trapped in fiber or surface gaps), or moisture-induced stickiness. They did not undergo chemical reactions with the substrate and were classified as physically adhering stains. Rust, however, was the product of the chemical reaction (oxidation) between iron (Fe), oxygen, and water, primarily forming iron (III) oxide (Fe_2_O_3_). Additionally, rust not only adhered to the surface but also penetrated deep into the porous structure of textiles, and even underwent chemical interactions (e.g., chelation and adsorption) with textiles. It was thus classified as a chemically transformed stain, making it more challenging to clean. In contrast, the mixed solid (sand, clay, and rust) stains incorporate stains with relatively weaker binding forces and larger particles (sand and clay), which effectively hinders the penetration and surface deposition of rust molecules. Consequently, its cleaning efficiency was slightly higher than pure rust but significantly lower than that of sand or clay alone. Ink had an extremely complex composition (simultaneously containing pigments, solvents, resins, and additives) and exhibited strong penetration into the fiber interior. Once cured, ink formed a tough film that encapsulated pigments and adhered firmly to the fibers, rendering them difficult to dissolve and becoming prone to bleeding/smudging. Thus, ink stains presented significant cleaning challenges. Synthetic blood stains primarily consisted of water, water-soluble red dyes (food coloring, acid dyes, etc.), thickeners (e.g., corn syrup, carboxymethyl cellulose, and carbomer), and preservatives. Cleaning agents readily dispersed its pigments, removed sugars and thickeners, and destroyed its chromophore (porphyrin ring), making it relatively easy to clean. Oil mainly consisted of grease physically adhering to textile surfaces. In addition, oil was insoluble in water but readily soluble in organic solvents or emulsified by surfactants. The oil stain used in this study was aged oil, which had penetrated deeper into the fibers and, over time, had oxidized, polymerized, or adsorbed dust, transforming into a viscous, insoluble, and stubborn stain, thus presenting a moderately higher cleaning difficulty. The mixed liquid stains, composed of ink, synthetic blood, and liquid lard, combined the adhesion mechanisms of various stains, resulting in the greatest cleaning difficulty.

### 2.3. Mechanical Strength Analysis of Stained Textiles Before and After Cleaning

Table 2 presents the mechanical strength of stained textiles before and after cleaning with various methods. As shown in Table 2, the mechanical strength of stained textiles was slightly lower than that of textiles without absorbing stains, indicating that the infestation of stains, even for a short time, also reduced the mechanical performance of textiles. This occurred because stains deposited on textile surfaces penetrated deeply into the fibers, causing the corrosion or decomposition of internal fibers, and then resulted in softening, dampness, stickiness, and diminishing mechanical strength of textiles. Furthermore, textiles with adhered stains were also more prone to breeding mold/bacteria and other microorganisms. In turn, these microorganisms generated heat, organic acids, and enzymes during their metabolic processes. This further accelerated damage to the textiles/fibers, leading to a decline in mechanical strength, and ultimately resulting in accelerated deterioration and a shortened lifespan. Moreover, this conclusion further confirmed the necessity of stain removal in textile relics to prolong their lifespan. In addition, by comparing the mechanical strength of stained textiles before and after the cleaning treatment, it was observed that, regardless of the stain type or cleaning methods, the mechanical strength of textiles after cleaning treatments did not decrease significantly; rather, it was relatively stable and even showed a slight upward trend. This was because the cleaning methods used in this study did not involve strong mechanical forces or agitation, instead belonging to relatively mild/non-mechanical cleaning approaches. Consequently, regardless of the cleaning methods applied, no significant decrease in tensile strength was observed. In addition, this finding also suggests that the proposed cleaning methods do not degrade the mechanical properties of textile relics, thus confirming their safety and efficacy.

### 2.4. EDS Analysis of Stained Textile Relics Before and After Cleaning

**Figure 3:**
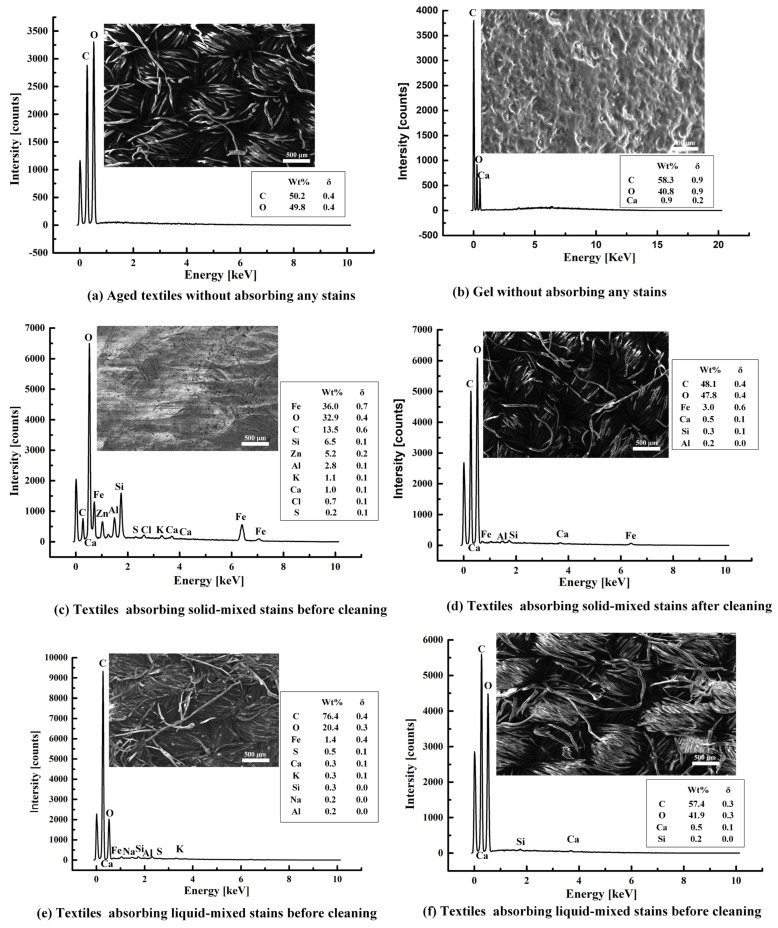
EDS of textiles absorbing mixed solid/liquid stains before and after the developed optimal cleaning method (G+U) used in this work.

Figure 3 illustrates the energy-dispersive spectra of aged textiles, gels, and textiles absorbing mixed solid/liquid stains before and after cleaning with the optimal method (G+U). As illustrated in Figure 3, the aged cotton textile mainly contained C (50.2%) and O (49.8%). The gel mainly contained C (58.3%), O (40.8%), and Ca (0.9%). The textiles adsorbing mixed solid stains mainly contained Fe, O, C, Si, Zn, Al, K, Ca, Cl, and S. The textiles adsorbing mixed liquid stains mainly contained C, O, Fe, S, Ca, K, Si, Na, and Al. However, after cleaning with the developed optimal method (G+U) in this work, no matter what kind of stain, most of impurities on the surface of the stained textiles were not only basically removed, but no new elements were introduced after cleaning with the developed optimal method. This demonstrates that the optimal method developed in this work has the advantages of an excellent cleaning effect and outstanding safety, and thus is suitable for cleaning stains on the surface of textile relics. Specifically, after the G+U cleaning treatment, the impurities Fe, Si, Zn, Al, Ca, K, Cl, and S in the simulated samples of textile relics adsorbing mixed solid stains were basically removed. Among them, Zn, K, Cl, and S were removed completely, and the content of Fe, Ca, Si, and Al were also obviously reduced. the contents of C and O elements also returned to normal levels. Meanwhile, the impurities Fe, S, Ca, K, Si, Na, and Al in the simulated samples of cotton textile relics adsorbing mixed liquid stains were basically removed after cleaning with the optimal method. Among them, after the G+U cleaning treatment, Fe, S, K, Na, and Al were removed completely, while the content of Ca and Si was obviously reduced, and the content of C and O elements also returned to normal levels.

Moreover, Figure 3 also reveals that mixed solid stains primarily deposit onto textiles in a particulate form; however, after the cleaning treatment, the surface particulate matter was significantly reduced, and the fibers regained a smooth surface morphology. In contrast, mixed liquid stains predominantly penetrated into the interior of the fibers. Consequently, minimal morphological changes were observed before and after cleaning, with the primary differences being elemental variations. This further validated that it was unreasonable to assess the cleaning efficacy based solely on a single indicator; therefore, a comprehensive evaluation integrating multiple criteria was essential. Furthermore, this finding not only confirmed the distinct adsorption modes and inherent complexities of different stains on substrates, but also substantiated the necessity and scientific rationale for conducting research on cleaning textile relics contaminated with diverse stain types. Additionally, it was found that the gel prepared in this study had the typical structural characteristics of hydrogels, such as abundant pore channels and small pore diameters.

**Figure 4:**
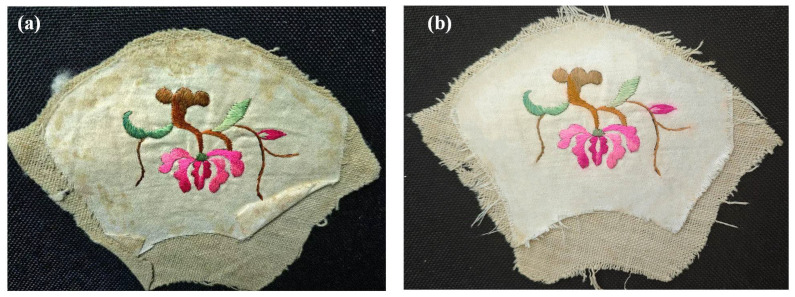
Practical application of optimal method in textile relics: (**a**) uncleaned; (**b**) after cleaning.

Furthermore, to validate the effectiveness of the developed cleaning method, the developed optimal cleaning method (G+U) in this work was also applied to the natural-colored silk Miao embroidery piece. The result showed that, after the cleaning treatment with the developed optimal cleaning method (G+U) used in this work, its appearance was significantly cleaner compared to before the cleaning (as shown in Figure 4). This once again confirms that the cleaning method we proposed is safe and efficient, and holds promise for application in cleaning contaminated textile artifacts.

### 2.5. Analysis of In-Situ Cleaning Mechanism

**Figure 5:**
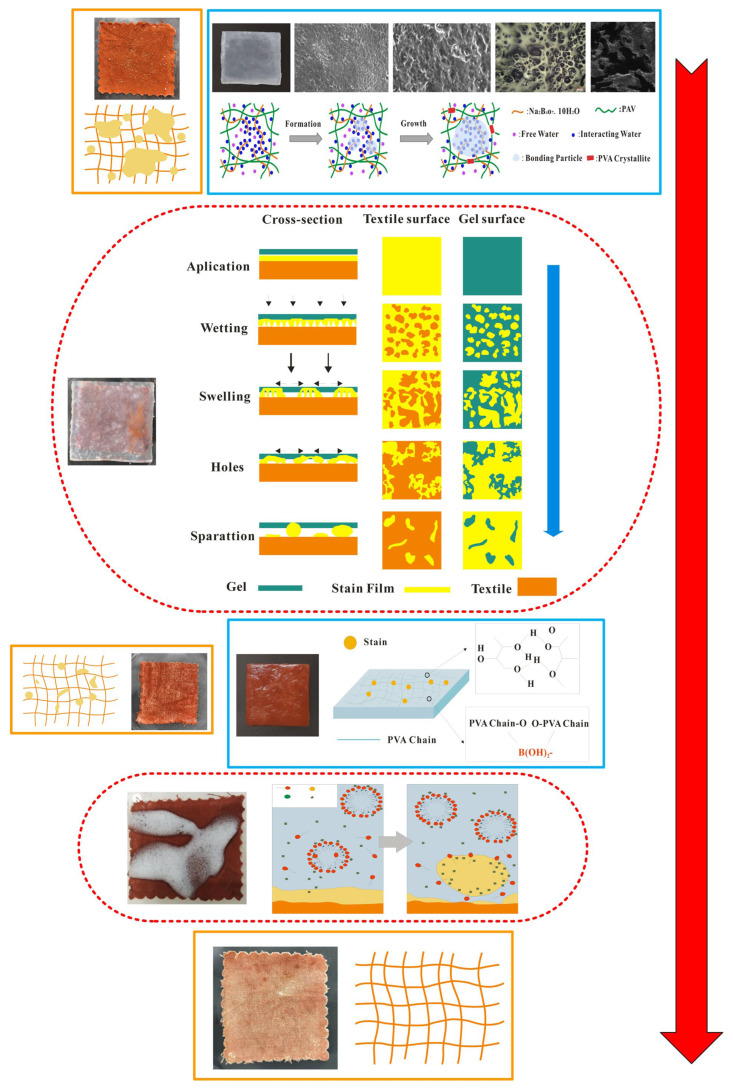
The cleaning process and mechanism of G+U cleaning method.

Figure 5 clearly illustrates the cleaning process and mechanism of the G+U cleaning method. As shown in Figure 5, the G+U cleaning method primarily consisted of two cleaning stages: hydrogel application and ultrasonic emulsification. During the hydrogel application stage, a soft/deformable hydrogel capable of conforming tightly to the complex surface contours of textile relics and featuring a multidimensional network structure with multiscale macropores was placed onto textiles (yarns/fibers) laden with accumulated particulate deposits. Utilizing its macro-porous, multidimensional network structure, the hydrogel dissolved or loosened contaminants (such as ions, small molecules, and microparticles) within its physical network or adsorbed them onto its polymer chains. This process effectively prevents the redisposition of stains onto the relic’s surface or their migration to other areas. Subsequently, when hydrogel was removed, the contaminants contained within its multidimensional space were simultaneously carried away (see Figure 5).

**Figure 6:**
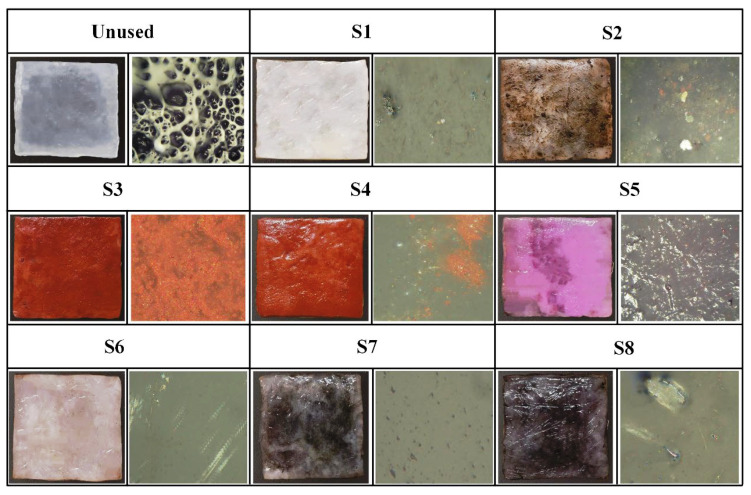
Appearance morphology of gel before and after use under G +U cleaning condition.

As shown in Figure 6, the original gel exhibited a semi-translucent, milky-white, gelatinous appearance with a uniform texture and smooth surface. Significant morphological changes occurred after its application as a cleaning material onto the stained textiles. In particular, the gel applied to the sand-stained textile appeared grayish-yellow. The gel applied to the clay-stained textile developed a grayish-brown hue with substantial particle adhesion across its surface, and exhibited localized cracking along the edges due to particle compression. The gel applied to the rust-soiled textiles displayed a uniform deep reddish-brown color. The gel applied to the mixed-solid-stain-soiled textiles showed a mottled color pattern with embedded particles and partial surface cracking. The gel applied to the blood-stained textile presented an uneven rosy pink color. The gel applied to the ink-stained cloth became completely opaque, with pure black coloration in the central region, while the peripheral areas developed an uneven dark halo effect. The gel applied to the oil-stained textiles produced an oily sheen on the surfaces. The gel applied to the mixed-liquid-stain-soiled textiles appeared predominantly black with interspersed oil spots and an overall cloudy appearance.

Clearly, the poultice cleaning method of gel belongs to a typical approach that utilizes the porous network physical structure of gel to achieve the targeted removal of contaminants from cultural relic surfaces. Furthermore, the cleaning process acts only on the specific surface area requiring cleaning and does not penetrate into the substrate’s interior or adjacent areas that do not require cleaning. Thus, the poultice cleaning method of gel effectively avoids the uncontrolled flow and penetration of cleaning agents onto the relic’s surface or within its pores, thereby maximizing the protection of the artifact itself. Specifically, the process of the poultice cleaning method of gel mainly includes gel system contacts with stained textiles. Water within the gel is slowly released, wetting and softening the stain layer. Cleaning agents slowly diffuse out of the gel network to the gel–artifact interface, where the active ingredients of the cleaning agent undergo chemical reactions (such as chelation, complexation, solubilization, or enzymatic breakdown) with the target stains. The reaction products (dissolved ions/molecules and loosened particles) are confined or adsorbed within the gel network. Upon reaching the predetermined time/effect, the gel containing the dissolved/softened stain particles that are trapped/adsorbed by the network is carefully peeled off or removed as an intact piece from the artifact surface. Additionally, morphological images of the inner and outer layers of the gel prepared in this study reveal that the outer skin layer has few pores, small pores, and a relatively uniform pore size of approximately 0.5 μm. In contrast, the inner layer has more pores, larger pores, and a non-uniform pore size distribution, featuring a multilevel pore structure with both large and small channels. This structure also helps to explain how gel can effectively immobilize contaminants without leakage.

Ultrasonic emulsification cleaning is a synergistic cleaning method that utilizes the cavitation effect of ultrasound to provide powerful physical detachment and mass transfer driving forces for contaminant removal. It simultaneously leverages the unique solubilization capacity and low interfacial tension of emulsions to achieve efficient and gentle dissolution, emulsification, dispersion, and removal of contaminants. The process primarily involves the following stages. The emulsion system contacts the artifact, wetting the adsorbed contaminants and causing them to swell. This reduces the contact area between the contaminant and the substrate and promotes aggregation. When contaminant aggregation reaches a critical point, fractures and pore formation occur within the contaminant mass, further decreasing its contact area with the substrate. Once the contaminant–substrate contact area diminishes to a critical threshold, the contaminant then reorganizes into droplet-like forms on the substrate surface. Ultrasonic cavitation then facilitates their detachment from the substrate. Concurrently, the intense agitation and micro-jets generated by ultrasonic cavitation significantly accelerate the diffusion, penetration, and mass transfer of the emulsion within the contaminant layer and across the artifact surface. This dramatically enhances the solubilization and dissolution efficiency. The nanostructure of the emulsion, particularly the presence of the oil phase, absorbs and dissipates a portion of the cavitation energy. This mitigates the risk of potential mechanical damage to fragile artifact substrates (especially organic materials like paper, textiles, and painted layers) caused by intense cavitation, rendering the process gentler and more controllable than ultrasonic cleaning using pure water or solvents alone. Furthermore, the ultrasonic cavitation effect primarily occurs at the solid–liquid interface (contaminant/artifact surface), concentrating the energy precisely where cleaning is required, enabling localized and targeted cleaning.

In summary, the G+U cleaning method proposed in this study is a typical in-situ cleaning approach that utilizes the multidimensional physical network structure of gel combined with the chemical action of emulsion and the effects of ultrasonic cavitation. Meanwhile, the G+U cleaning method controls the release of surface contaminants from artifacts and their precisely targeted removal. Therefore, this G+U method helps maximize the protection of fragile artifact substrates against mechanical damage and chemical penetration. Additionally, the G+U cleaning method fully aligns with the conservation goals of maximizing substrate protection and minimizing intervention, making it particularly suitable for cleaning fragile, intricate artifacts or those bearing complex mixtures of contaminant types.

## 3. Conclusions

Removing dirt and failed reinforcement materials from the surface of textile relics is a complex and significant task in the field of textile heritage conservation. Regrettably, existing methods (e.g., manual scrubbing and ultrasonic cleaning) face challenges such as imprecise control over cleaning areas and uncontrolled diffusion of solvents/dissolved dirt into absorbent textile relics. Moreover, these imperfections lead to the secondary contamination or damage of the artifacts, and even the irreversible loss of cultural heritage information. To overcome the problems of the uncontrollable diffusion of cleaning solvents and secondary contamination from dissolved stains on textile relics, here, taking cotton textiles as a typical example, we systematically investigated the relationship between cleaning efficacy against eight types of stains (including sand, clay, and rust) and four in-situ methods (blank gel poultice, cleaning gel poultice, ultrasonic emulsification cleaning, and the synergistic cleaning method of cleaning gel poultice + ultrasonic emulsification).

The results indicated that, regardless of the stain type, the synergistic cleaning method (G+U: cleaning gel poultice + ultrasonic emulsification) consistently outperformed the other three methods tested (blank gel poultice, cleaning gel poultice alone, and ultrasonic emulsification alone). This is because the synergistic method is a typical cleaning approach that utilizes the physical structure of the gel’s porous network combined with the chemical action of the emulsion’s wetting/dissolving power to achieve the targeted removal of contaminants from the artifact’s surface. Simultaneously, it was also found that the gel loaded with cleaning agents exhibited significantly superior performance compared to the blank (unloaded cleaning agents) gel. This is because the blank gel (unloaded cleaning agents) can only exert the adsorption effect of gel material, whereas the cleaning gel (loaded cleaning agents) leverages both the gel’s adsorption and the chemical action of active ingredients in cleaning agents. Therefore, the cleaning effect of the blank gel is inferior to that of the cleaning gel. Furthermore, this conclusion also demonstrates that the gel primarily acts as a carrier for the cleaning fluid. While restricting the free flow of the cleaning liquid, it ensures sufficient contact between its active ingredients and the pollutants, thereby enabling the efficient, controlled, and in-situ removal of dirt. Consequently, to adapt this method for the targeted removal of specific stains from textile relics in the future, one only needs to load the gel with a cleaning agent matched to the type of stain adhered to the artifact for efficient stain removal. Additionally, comparing the cleaning efficacy for different stains revealed that the poultice methods (both blank and cleaning gels) were more suitable for removing solid stains, while the ultrasonic emulsification method was better suited for liquid stains. Therefore, in subsequent practical conservation work on real artifacts, the optimal cleaning method should be selected based on the actual state of stains on cultural relics. Moreover, based on the analysis of appearance, whiteness, tensile strength retention rate, and EDS data, it was also found that the proposed G+U (cleaning gel + ultrasonic emulsification) synergistic cleaning method not only efficiently and safely removed various types of stains, but also maximally preserved the fragile artifact substrate from mechanical damage and chemical penetration damage. Thus, it falls under the category of safe, efficient, and in-situ cleaning methods as defined in the field of cultural heritage conservation. Therefore, it holds promise for application in cleaning fragile, complex artifacts or those containing multiple types of dirt, especially textile relics. Moreover, this study solely focused on the correlation between cleaning methods and stain removal efficacy without examining parameters like optimal cleaning settings for different stains, economic costs, or long-term impacts on artifacts. Hence, further research should address these aspects.

## 4. Material and Methods

### 4.1. Experimental Materials and Equipment

In order to develop efficient in-situ cleaning methods for stained textile relics, the following materials were utilized in the experimental study: cotton fabrics (100% cotton, plain weave, 248 × 269 threads/10 cm, 190 g/m^2^, 0.037 mm thick) were provided by Anhui Tuoyan Experimental Materials Co., Ltd. (Hefei, China); 1 mol/L HCl solution was bought from Shanghai Sinopharm—Shenzhen Boruianda Technology Co., Ltd. (Shenzhen, China); artificial synthetic blood was purchased from Fuzhou Feijing Biotechnology Co., Ltd. (Fuzhou, China); carbon inks were acquired from Shanghai M&G Stationery Co., Ltd. (Shanghai, China); iron rust powder was supplied by Hebei Wanli Evolution Industry Co., Ltd. (Shijiazhuang, China); sand and clay were obtained from Zhongjiang Park, Wuhu, Anhui; lard was purchased from Lianhua Supermarket, Wuhu, China; 98% alcoholysis degree Type 1799 polyvinyl alcohol (PVA) and 99.8% purity nano-silica were purchased from Bidepharm (Suzhou)Technology Co., Ltd. (Suzhou, China); 50 g/L of saturated borax solution was bought from Beijing Biotechnology Co., Ltd. (Beijing, China); bio-enzymes (99% trypsin, trypsin, and lipase) were bought from XiYa Reagent; lipase (≥100 units/mg) was bought from Hefei Basf Biotechnology Co., Ltd. (Hefei, China); 98% AR sodium dodecyl sulfate (SDS), 99.9% EDTA, sodium perborate, ascorbic acid, sophorolipid, tea saponin, sodium citrate, and glycerol were acquired from Shanghai Macklin Biochemical Technology Co., Ltd. (Shanghai, China); deionized water was prepared in the laboratory of Anhui Polytechnic University.

In addition, the following equipment was used during the experimental investigation: ultrasonic electric toothbrush (T100, Xiaomi, Beijing Jingdong Century Trading Co., Ltd., Beijing, China), pH test strips, magnetic stirrer (DF101S), thermostatic water bath shaker (GP-300C), electric stirrer (H2004G, Shanghai Meiyinpu Instrument Manufacturing Co., Ltd., Shanghai, China), electronic balance (YH-C30001, Shanghai Sunny Hengping Instrument Co., Ltd., Shanghai, China), fabric density meter (Y511B, Ningbo Dahe Instrument Co., Ltd., Ningbo, China), fabric thickness gauge (YG141, Changzhou Shuanggudunda Electromechanical Technology Co., Ltd., Changzhou, China), electronic ruler (YH-C30001, Shenzhen Shunyang Precision Engineering Electronic Technology Co., Ltd., Shenzhen, China), freeze-dryer (JY-DGJ0350, Shanghai Muni Laboratory Equipment Co., Ltd. Shanghai, China), digital camera (Sony ZV-1F, Beijing Jingdong Century Trading Co., Ltd., Beijing, China), intelligent digital whiteness meter (WSB-3A, Wenzhou Ludong Instrument Co., Ltd., Wenzhou, China), fabric strength tester (YG026HC), ultra-depth-of-field 3D microscope (KEYENCE VHX-S600E, Beijing Pinzhi Chuangsi Precision Instrument Co., Ltd., Beijing, China), and NexsG2 X-ray photoelectron spectrometer (Mercer Technology Co., Ltd., Terre Haute, IN, USA, Guoyi Quantum Technology (Hefei) Co., Ltd., Hefei, China).

### 4.2. Experimental Process and Design

To develop an in-situ, efficient, and controllable cleaning method suitable for fragile stained textile relics, the experiment was divided into the preparation of simulated samples of stained textile relics and the development of an efficient in situ cleaning method for stained textile relics. The detailed procedures are outlined as follows.

#### 4.2.1. Preparation of Simulated Samples of Stained Textile Relics

The preparation of substitute samples for stained textile relics was divided into fabric aging and stain contamination of aged samples. The specific procedure is listed as follows (see Table 3). First, fabric was cut into standard 5 cm × 5 cm swatches. These swatches were immersed in 1 mol/L hydrochloric acid solution at a liquor ratio of 1:30 for 7 h, with agitation every hour. After 7 h, the samples were removed, rinsed with deionized water until a neutral pH (rinsing water solution; pH = 7) was achieved, and air-dried at room temperature in the shade. Then, moderately aged samples with tensile strength retention rates of 10–20% were obtained. Subsequently, the aged samples were contaminated with eight types of stains {sand, clay, rust, mixed solids (sand–clay–rust), artificial blood, oil, ink, and mixed liquids (artificial blood–ink–oil)} according to contamination steps listed in Table 1. The contaminated samples were then stored in a constant temperature and humidity chamber (20 ± 2 °C, 65% RH ± 2%) for 30 days to maximize the authenticity of experimental results.

#### 4.2.2. Development Experiment of Efficient In-Situ Cleaning Method for Stained Textile Relics

To develop an efficient in-situ cleaning method for stained textile relics, blank gel (unloaded cleaning agent) poultice (G_b_)/cleaning gel (loaded cleaning agent) poultice (G)/ultrasonic emulsification (U_e_)/cleaning gel poultice + ultrasonic emulsification (G+U) cleaning methods were used in this study. Specific cleaning methods are provided in Table 4.

### 4.3. Experimental Testing Indicators and Methods

#### 4.3.1. Appearance Morphology

In order to investigate the relationship between cleaning methods and the cleaning effect of stained textile relics, the appearance morphology of a simulated samples of stained textile relics before and after the treatment of different cleaning methods was individually measured with the help of a digital camera (SONY ZV-1F) and ultra-depth-of-field 3D microscope (VHX-S600E).

#### 4.3.2. Washing Efficiency

In order to quantitatively evaluate the effect of different cleaning methods on the washing efficiency of stained textile relics, the whiteness of simulated samples of the stained textile relics before and after washing with different cleaning methods was tested at 5 spots using a whiteness meter (Data color 650) under the D65 light source, as specified in GB/T 7974-2013 [36]. The average whiteness value of stained textiles tested 5 times was taken as the final whiteness of the tested sample to ensure the stability and repeatability of the results. The washing efficiency was calculated using Equation (1), as follows:

(1) $$CR=\frac{R_{1}-R}{R_{0}-R}\times 100\%$$

where CR is the cleaning rate (%); *R*_0_ is the average whiteness of the aged textile before staining (the simulated samples of textile relics without absorbing stains); *R* is the average whiteness of the stained textiles before cleaning; $R_{1}\;$ is the average whiteness of the stained textiles after cleaning. Furthermore, it must be pointed out that the whiteness index here is scaled from 0 to 100.

#### 4.3.3. Mechanical Strength Test

To identify whether a drop in the strength of textile relics occurred after cleaning with different cleaning methods, the elongation at break of the simulated samples of stained textile relics before and after different cleaning treatments was tested using a fabric strength tester (YG026HC). Testing methods and operations refer to GB/T 3923.1-2013 [37] textiles, fabrics-tensile properties-part 1, determination of breaking strength and elongation at break (strip method). However, the sample dimensions and equipment parameters in this study were proportionally scaled down by a factor of 10 according to the national standard. Specifically, the effective width of each sample was 5 ± 0.1 mm (excluding selvage), and the length should meet a gauge length of 20 mm. The equipment parameters include a pretension of 1 N, a clamping distance of 20 mm, a moving speed of 50 mm/min, a fixed force of 1000 N, and a fixed elongation of 150%. Furthermore, to ensure data reliability, each sample was tested according to the direction of the warp and weft (5 warps and 5 wefts), respectively. The average value of five times in each direction was taken as the final value of the tensile strength of the sample in this direction. The influence of the cleaning treatment on the mechanical properties of the stained textiles was characterized by the retention rate of the breaking strength, which was calculated by the following formula:

$$∆T=\frac{T_{a}}{T_{0}}\times 100\%$$

where ∆T is the retention rate of the breaking strength (%); $T_{a}$ is the average breaking strength of the stained textiles after cleaning; $T_{0}$ is the average breaking strength of aged textiles.

#### 4.3.4. Energy-Dispersive Spectrometer (EDS)

In order to investigate whether the optimal cleaning method was suitable for the actual cleaning of precious textile relics, and to reveal its mechanism of action, the energy-dispersive spectra of the simulated samples of stained textile relics {absorbing mixed solids (sand–clay–rust) and mixed liquids (artificial blood–ink–oil)} before and after cleaning with the optimal cleaning methods were systematically tested with the help of the NexsG2 X-ray (Hefei, Chian)photoelectron energy-dispersive spectrometer (Mercer Technology Co., Ltd., Terre Haute, IN, USA). The power setting was 120 W, including monochromatic Al Ka, and the X-ray beam spot was 400 μm; the wide spectrum and high-resolution scanning energies were set at 100 eV and 30 eV, respectively.

## Figures and Tables

**Table 1:** Cleanliness of stained textiles before and after cleaning with different methods.

			S1	S2	S3	S4	S5	S6	S7	S8
Wv	R		63.27	49.84	23.68	29.89	34.46	69.96	21.17	22.69
	R1	G_b_	68.94	53.13	25.43	31.64	41.75	88.76	23.36	23.68
		G	73.23	58.45	27.26	33.66	46.35	87.27	28.59	25.94
		U_e_	60.44	44.99	20.21	23.05	73.54	86.88	47.69	44.91
		G+U	84.08	78.46	52.72	56.91	82.21	85.74	48.82	46.24
CD		G_b_	5.67	3.29	1.75	1.77	7.29	−1.2	2.19	0.99
		G	9.96	8.61	3.58	3.79	11.89	−2.69	7.42	3.25
		U_e_	−2.83	−4.85	−3.47	−6.84	39.08	−3.08	26.52	22.22
		G+U	20.81	28.62	29.04	27.02	47.75	−4.22	27.65	23.55
CR, %		G_b_	26.06	9.35	2.85	3.21	14.42	24.34	3.43	1.59
		G	45.77	24.47	5.84	6.87	23.51	54.56	11.62	5.21
		U_e_	−13.01	−13.78	−5.66	−12.40	77.28	62.47	41.53	35.64
		G+U	95.63	81.33	47.33	49.00	94.42	85.60	43.30	37.78

Note: $R_{0}$ is the average whiteness of the aged textiles without adsorbing any stains (85.03); R is the average whiteness of the stained textiles (the simulated samples of textile relics absorbing different stains) before cleaning; $R_{1}\;$ is the average whiteness of the stained textiles after cleaning; Wv is the whiteness value; CD is the whiteness difference; CR is the cleaning rate (%).

**Table 2:** Mechanical strength of stained textiles before and after cleaning with different methods.

Yarn Direction	Stain Type	Breaking Strength (N)					Retention Rate of Breaking Strength (%)			
		T_a_	T_b_							
			G_b_	G	U_e_	G+U	G_b_	G	U_e_	G+U
Warp	S1	9.31	9.28	9.19	9.23	9.24	99.15	98.18	98.61	98.72
	S2	9.27	9.26	9.27	9.26	9.25	98.93	99.04	98.93	98.82
	S3	9.29	9.27	9.25	9.28	9.26	99.04	98.82	99.15	98.93
	S4	9.15	9.16	9.22	9.21	9.27	97.86	98.50	98.40	99.04
	S5	9.23	9.21	9.18	9.17	9.18	98.40	98.08	97.97	98.08
	S6	9.28	9.19	9.18	9.26	9.16	98.18	98.08	98.93	97.86
	S7	9.29	9.26	9.22	9.27	9.23	98.93	98.50	99.04	98.61
	S8	9.14	9.17	9.27	9.21	9.16	97.97	99.04	98.40	97.86
Weft	S1	8.69	8.65	8.59	8.66	8.29	98.41	97.72	98.52	94.31
	S2	8.58	8.57	8.56	8.58	8.56	97.49	97.38	97.61	97.38
	S3	8.82	8.75	8.79	8.58	8.76	99.54	100	97.61	99.65
	S4	8.47	8.48	8.43	8.46	8.45	96.47	95.90	96.24	96.13
	S5	8.76	8.73	8.74	8.74	8.73	99.32	99.43	99.43	99.32
	S6	8.77	8.74	8.71	8.75	8.72	99.43	99.09	99.54	99.20
	S7	8.73	8.69	8.59	8.75	8.71	98.86	97.72	99.54	99.09
	S8	8.67	8.68	8.73	8.29	8.65	98.75	99.32	94.31	98.41

Note: $T_{a}$ is the average breaking strength of the stained textiles after cleaning; $T_{b}$ is the average breaking strength of the stained textiles before cleaning; $T_{0}$ is the average breaking strength of aged textiles before absorbing stains (warp = 9.36 N; weft = 8.79 N).

**Table 3:** The preparation process of substitute samples for stained textile relics.

Stage	Aged Steps		Process Diagram
Aged stage	First, fabric was cut into standard 5 cm × 5 cm swatches. These swatches were immersed in a 1 mol/L hydrochloric acid solution at a liquor ratio of 1:30 for 7 h, with agitation every hour. After 7 h, the samples were removed, rinsed with deionized water until a neutral pH (pH = 7) was achieved, and air-dried at room temperature in the shade. Then, moderately aged samples with tensile strength retention rates of 10–20% were obtained.		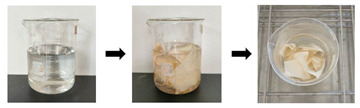
Stain contamination stage	Stain type	Stain contamination steps	Process diagram
	Sand stain (S1)	First, the artificially aged samples were laid flat on the workbench and treated with deionized water to achieve a moist state with a 20% moisture content. Secondly, sandy soil was uniformly sprinkled onto the surface of the moist sample at a mass ratio of 10:1 (sandy soil to textile). To ensure even distribution of the sandy soil, a spatula was used to gently spread and press it. Finally, the moist samples with absorbed sandy soil were air-dried at room temperature for 48 h to allow the stains to fully adhere, yielding substitute samples of textile relics with adsorbed sand stains.	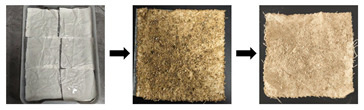
	Clay stain (S2)	First, the artificially aged samples were placed flat on the workbench and treated with deionized water to achieve a moist state with a 20% moisture content. Secondly, clay (passed through a 16-mesh sieve) was uniformly sprinkled onto the surface of the moist samples at a mass ratio of 10:1 (clay to textile). To ensure even distribution, a spatula was used to gently spread and press the clay. Finally, the moist samples with absorbed clay were left to stand at room temperature for 48 h to ensure full adhesion of the stains, thus obtaining substitute samples of textile relics with adsorbed clay stains.	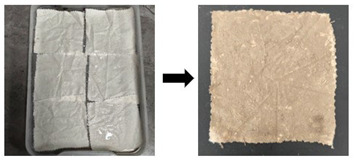
	Rust stain (S3)	First, artificially aged samples were placed flat on the workbench and treated with deionized water to achieve a moist state with a 20% moisture content. Next, iron rust was uniformly sprinkled onto the surface of the moist samples at a mass ratio of 10:1 (clay to textile). To ensure even distribution of the clay, a spatula was used to gently spread and press it. Finally, the moist samples with absorbed iron rust were left at room temperature for 48 h. Deionized water was sprayed every 12 h to ensure full adhesion of the stains, thus obtaining substitute samples of textile relics with adsorbed iron rust stains.	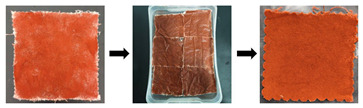
	Mixed solid stain (S4)	First, artificially aged samples were placed flat on the workbench and treated with deionized water to achieve a moist state with a 20% moisture content. Next, the mixed solids (4 sand/4 clay/2 rust) were uniformly sprinkled onto the surface of the moist samples at a mass ratio of 10:1 (mixed solids to textile). To ensure even distribution of the mixed solids, a spatula was used to gently spread and press them. Finally, the moist samples with absorbed mixed solids were left to stand at room temperature for 48 h. Deionized water was sprayed every 12 h to ensure sufficient adhesion of the stains, thus obtaining substitute textile relic samples with adsorbed mixed solid stains.	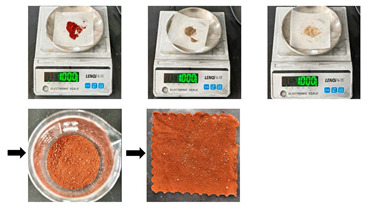
	Blood stain (S5)	First, aged samples were placed flat on a horizontal workbench. Then, a rubber-tipped dropper was used to evenly apply 1 mL of the blood stain solution (diluted at a 1:1 ratio with deionized water and artificial blood) onto the sample surface. Finally, the samples were left to absorb the blood stains at room temperature for 48 h, thus obtaining a substitute sample of textile relics with adsorbed blood stains.	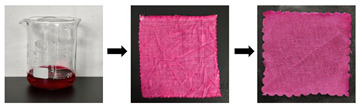
	Oil stain (S6)	First, aged samples were placed flat on a horizontal workbench. Then, a rubber-tipped dropper was used to evenly apply 1 mL of lard onto the sample surface. Finally, the samples were left to absorb the lard at room temperature for 48 h, thus obtaining a substitute sample of textile relics with adsorbed oil stains.	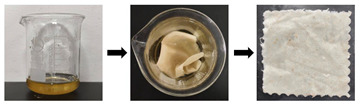
	Ink stain (S7)	First, aged samples were placed flat on a horizontal workbench. Then, a rubber-tipped dropper was used to evenly apply 1 mL of the ink-stained solution (diluted at a 5:1 ratio of deionized water to ink) onto the sample’s surface. Finally, the samples were allowed to absorb the ink at room temperature for 48 h to obtain a substitute sample of textile relics with adsorbed ink stains.	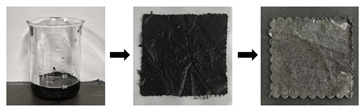
	Mixed liquid stain (S8)	First, aged samples were placed into a mixed liquid stain solution prepared at a 1:3 ratio of deionized water to a blend of blood, ink, and lard (1:1:1). The samples were submerged for 30 min, then removed and air-dried at room temperature for 48 h. This yielded substitute samples of textile relics with adsorbed mixed liquid stains.	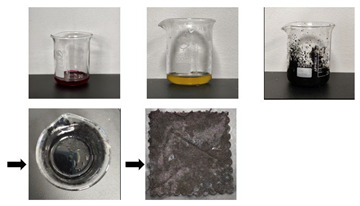

**Table 4:** Detailed process of cleaning methods.

Cleaning Method	Description of Specific Cleaning Methods
G_b_	Firstly, add 10% PVA powder to deionized water, stir and heat to 90 °C, and continue stirring until completely dissolved (10 min). Then, stop heating and cool to room temperature (30 min). Slowly drip 1% borax solution into PVA solution to form a precursor gel. Additionally, to ensure sufficient cross-linking and curing, place the obtained precursor gel in a freezer for 3 h, and then remove it to thaw at room temperature, thus obtaining a blank gel. Apply the obtained blank gel directly onto the surface of the contaminated textiles; then, place a 5 N weight on top of the textiles and maintain it for 10 min to ensure uniform adhesion to the stained textiles. Use tweezers to peel off the gel, repeating the pressing and peeling actions 10–15 times until the blank gel loses its adhesion to the stains. Furthermore, to enhance the gel’s adhesiveness during the cleaning process, each time gel is peeled off, use a garment steamer to spray steam for 10 s from a distance of 10 cm to soften the gel. 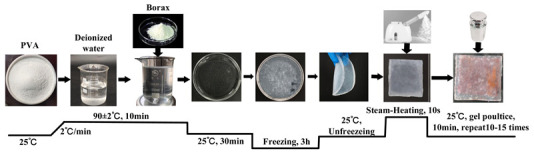
G	Firstly, add 10% PVA powder to deionized water, stir and heat to 90 °C, and continue stirring until completely dissolved (10 min). Then, stop heating and cool to room temperature (30 min). Then, add the cleaning agent at a mass ratio of 1 (cleaning agent):20 (contaminated fabric), stir for 5 min, and slowly drip a 1% borax solution to form a precursor gel. Additionally, to ensure sufficient cross-linking and curing, place the obtained precursor gel in a freezer for 3 h, and then remove it to thaw at room temperature, thus obtaining a cleaning gel. Apply the obtained cleaning gel directly onto the surface of the contaminated textiles; then, place a 5 N weight on top of the textiles and maintain it for 10 min to ensure uniform adhesion to the stained textiles. Use tweezers to peel off the gel, repeating the pressing and peeling actions 10–15 times until the cleaning gel loses its adhesion to the stains. Furthermore, to enhance the gel’s adhesiveness during the cleaning process, each time gel is peeled off, use a garment steamer to spray steam for 10 s from a distance of 10 cm to soften the gel. 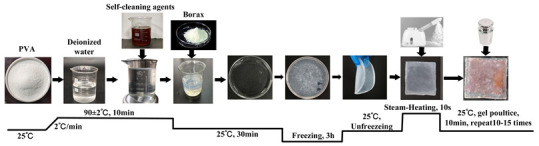
U_e_	Firstly, prepare a cleaning solution at a mass ratio of 1 (detergent):20 (deionized water), and add the solution to emulsification equipment via an infusion pump, forming a cleaning unit with the soiled cloth placed in the cleaning tank. Then, perform the cleaning using an ultrasonic vibrating brush. During the process, place a superabsorbent polymer pad under the stained textile to absorb wastewater, preventing backflow and recontamination. Set the ultrasonic vibration frequency to 20,000 Hz and maintain a reciprocating rate of 30 cycles/min for 10 min to complete the treatment. 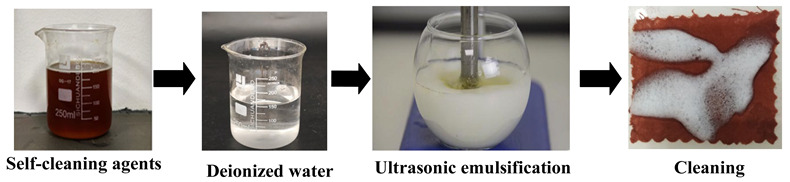
G+U	First, use the cleaning gel poultice cleaning method to remove loose stains from the surface of the stained textiles. Then, perform the improved ultrasonic emulsification cleaning treatment to perform emulsification cleaning for slits, interior areas, and stubborn stains. 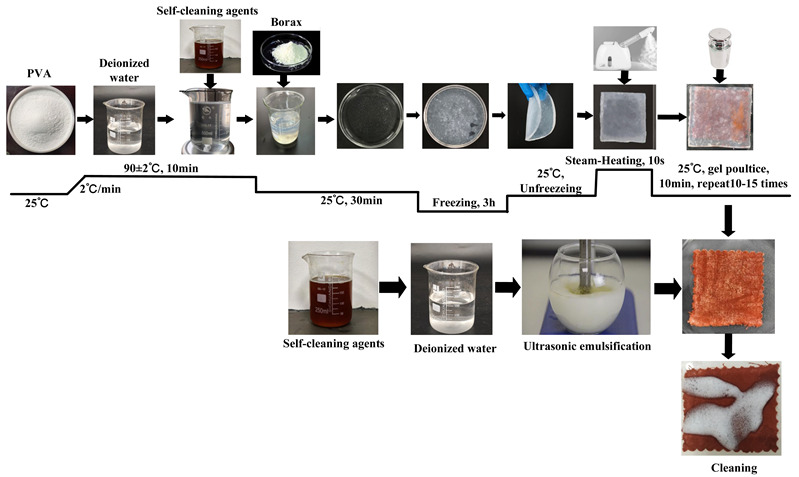

Note: Formulation of cleaning agents used in this study was composed of 2% tea saponin, 2.5% sophorolipid, 3% ascorbic acid, 1.5% sodium perborate, 0.6% lipase, 2% nano-silica, 2.5% EDTA, and 2% sodium citrate (adjusted to pH ≈ 7).

## Data Availability

The datasets generated during the current study are available from the corresponding author on reasonable request.
